# Total laparoscopic nephroureterectomy for upper urinary tract urothelial carcinoma under a single surgical position

**DOI:** 10.1186/s12957-019-1601-0

**Published:** 2019-04-11

**Authors:** Xuebao Zhang, Ke Wang, Jiajia Ma, Qiqiang Zhang, Chu Liu, Yuanshan Cui, Chunhua Lin

**Affiliations:** 1 Department of Urology, The Affiliated Yantai Yuhuangding Hospital of Qingdao University, NO. 20 East Yuhuangding Road, Yantai, 264000 Shandong China; 2 Department of Urology, The Affiliated Hospital of Qingdao University, Qingdao, Shandong China

**Keywords:** Single position, Total laparoscopy, Upper urinary tract urothelial carcinoma

## Abstract

**Background:**

To assess the feasibility and effectiveness of total laparoscopic nephroureterectomy for upper urinary tract urothelial carcinoma (UUTUC) under a single surgical position.

**Methods:**

The medical data of 89 UUTUC patients were collected, who were treated in our institution from Jan 2016 to Jun 2018. The 45 cases that underwent total laparoscopic nephroureterectomy with a single position were allocated in the test group, while the 44 patients who received retroperitoneal laparoscopy combined with hypogastric oblique incision were assigned in the control group. We compared the two groups in perioperative indicators and tumor recurrence rate and analyzed the clinical effect of the new surgical treatment of UUTUC.

**Results:**

All 89 operations for UUTUC were successful and had no conversion to open surgery. No obvious complications occurred during the perioperative period. The test group had significantly shorter average operation time (96.58 ± 8.56 min versus 147.45 ± 9.16 min), less blood loss (39.58 ± 4.15 ml versus 46.50 ± 4.58 ml), earlier ambulation (7.47 ± 1.01 h versus 11.39 ± 1.82 h), and shorter length of stay in hospital (6.98 ± 1.14 days versus 9.89 ± 1.51 days) (*P* < 0.05). The visual analogue scale (VAS) scores of the test group at 1 h, 12 h, and 24 h after operation were lower compared with those of the control group (*P* < 0.05). No significant difference was found in the tumor stage, tumor grade, postoperative gastrointestinal function recovery time, follow-up time, and tumor recurrence rate between the two groups.

**Conclusions:**

Compared with the traditional surgical methods, the total laparoscopic treatment of UUTUC under a single surgical position had advantages of shorter operation time, less blood loss, and early postoperative ambulation. The new operative method could shorten the length of stay and accelerate recovery of patients, and it is a viable surgical procedure which deserved clinical application and promotion.

**Trial registration:**

Our trial was approved and has been registered in the ethics committee of the Yantai Yuhuangding Hospital (Approval NO.[2015]171).

## Background

Upper urinary tract urothelial carcinoma (UUTUC) is a rare kind of urinary tumor, with gross hematuria as its common initial symptom [1]. Most patients were finally diagnosed with UUTUC for occasional macroscopic hematuria. Laparoscopic surgery is one of the main surgical solutions for UUTUC [2, 3]. Retroperitoneal laparoscopic nephroureterectomy plus transurethral electric resection of the bladder cuff has been widely applied for treating UUTUC [4, 5]. In recent years, studies have shown that retroperitoneal laparoscopy combined with hypogastric oblique incision has more obvious advantages [6, 7].

After analyzed and summarized the operation method and the clinical effects of UUTUC, our institute has developed a new surgical method for the treatment of UUTUC—total laparoscopic nephroureterectomy under a single surgical position based on the anatomy of the upper urinary tract and the therapeutic principles of UUTUC. In this study, the effectiveness of this new method was studied in comparison with that of the conventional method in which the change of position was required during the operation. We confirmed that this new method could reduce surgical trauma and promote faster recovery of patients.

## Methods

### Clinical data

Our research has been approved by the Ethics Committee of Yantai Yuhuangding Hospital, and we have obtained the informed consent from all patients. And our research has been registered. Eighty-nine patients with UUTUC admitted to our institution from Jan 2016 to Jun 2017 were selected. Patients who underwent complete laparoscopic treatment of UUTUC were included in the experimental group, and patients who underwent retroperitoneal laparoscopy combined with hypogastric oblique incision were included in the control group.

**Fig. 1:**
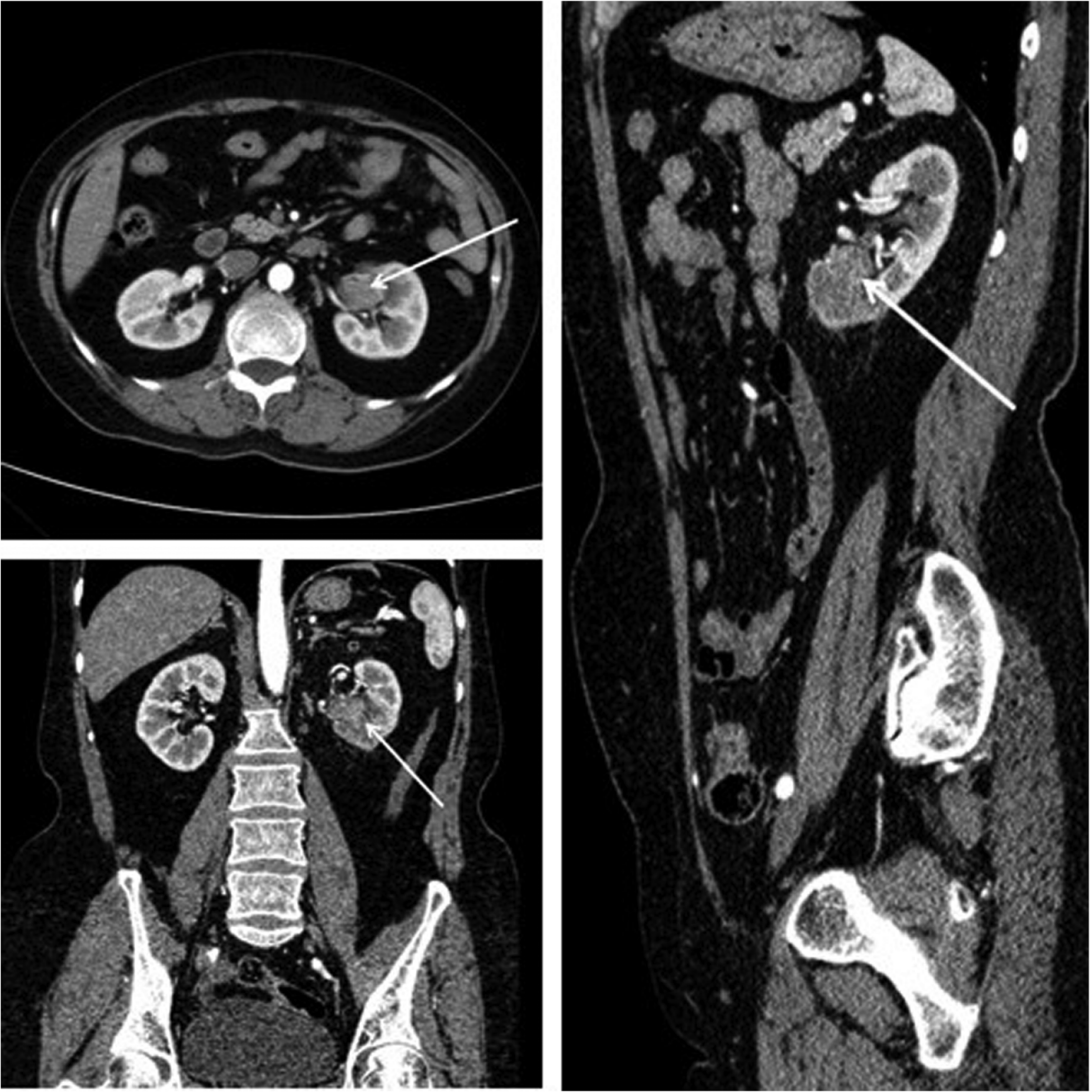
Determination of the tumor location by CT. Arrows indicate an upper urinary tract urothelial carcinoma

There were 45 patients in the test group, including 24 males and 21 females, 19 with left-sided tumors and 26 with right-sided tumors, and 31 with renal pelvic cancers while 14 with ureteral cancers. There were 44 patients in the control group, including 23 males and 21 females. The tumor location of 21 patients was on the left side while 23 patients were on the right side. There were 29 patients with renal pelvis cancer and 15 patients with ureteral cancer. All patients underwent computed tomographic urography (CTU) or magnetic resonance urography (MRU) before surgery to determine the tumor location (Fig. 1). Renal dynamic imaging and glomerular filtration rate (GFR) tests were performed to assess contralateral renal function. Thoracic and abdominal CT was performed to exclude tumor metastases, and cardiopulmonary function was assessed to exclude surgical contraindications. Visual analogue scale (VAS) was used to evaluate the degree of pain at 1 h, 12 h, 24 h, and 48 h after surgery. There were no statistically significant differences in the general conditions between the two groups, such as age, gender, tumor location, tumor stage, and grade (*P* > 0.05, Table 1).

**Table 1:** Characteristics and outcomes of the test and control group

	Test group (*n* = 45)	Control group (*n* = 44)	Statistics	*P* value
Age (years) (*x̅* ± *s*)	65.80 ± 6.06	65.68 ± 3.13	*t* = 0.116	0.908
Gender (M/F) (*n*)	24/21	23/21	*χ*^2^ = 0.010	0.920
Tumor (right/left) (*n*)	19/26	21/23	*χ*^2^ = 0.272	0.602
Tumor location (pelvis/ureter) (*n*)	31/14	29/15	*χ*^2^ = 0.090	0.764
Tumor stage (T_1_/T_2_/T_3_) (*n*)	21/17/7	20/18/6	*χ*^2^ = 0.119	0.942
Tumor grade (G_1_/G_2_/G_3_) (*n*)	16/23/6	14/25/5	*χ*^2^ = 0.296	0.862
Operation time (min) (*x̅* ± *s*)	96.58 ± 8.56	147.45 ± 9.14	*t* = − 27.112	< 0.01*
Intraoperative blood loss (ml) (*x̅* ± *s*)	39.58 ± 4.15	46.50 ± 4.58	*t* = − 7.475	< 0.01*
Postoperative pain score (*x̅* ± *s*)				
1 h after surgery	4.40 ± 1.23	5.18 ± 1.57	− 2.612	< 0.05*
12 h after surgery	2.33 ± 0.95	3.64 ± 1.35	− 5.273	< 0.01*
24 h after surgery	1.64 ± 1.07	2.18 ± 1.26	− 2.169	< 0.05*
48 h after surgery	1.22 ± 0.90	1.39 ± 0.95	− 0.838	0.404
Recovery of bowel function (h) (*x̅* ± *s*)	6.47 ± 1.08	6.39 ± 1.06	*t* = 0.354	0.724
Postoperative ambulation (h) (*x̅* ± *s*)	7.47 ± 1.01	11.39 ± 1.82	*t* = − 12.514	< 0.01*
Length of stay (days) (*x̅* ± *s*)	6.98 ± 1.14	9.89 ± 1.51	*t* = − 10.265	< 0.01*
Follow-up (month) (*x̅* ± *s*)	25.95 ± 5.49	25.90 ± 5.44	*t* = − 0.040	0.968
Tumor recurrence (*n*)				
Intravesical tumor recurrence	1	3	—	0.361
Extra-bladder implantation recurrence	0	2	—	0.242
Renal pelvic tumor recurrence	0/31	2/29	—	0.229
Ureteral tumor recurrence	1/14	3/15	—	0.598

*Statistically significant

### Excluding criteria

The excluding criteria were the following: patients with bilateral tumor, tumor with node metastasis or bladder carcinoma, adenocarcinoma and squamous cell carcinoma, simultaneous pelvis, and ureter tumor.

### Surgical procedures

#### Test group

**Fig. 2:**
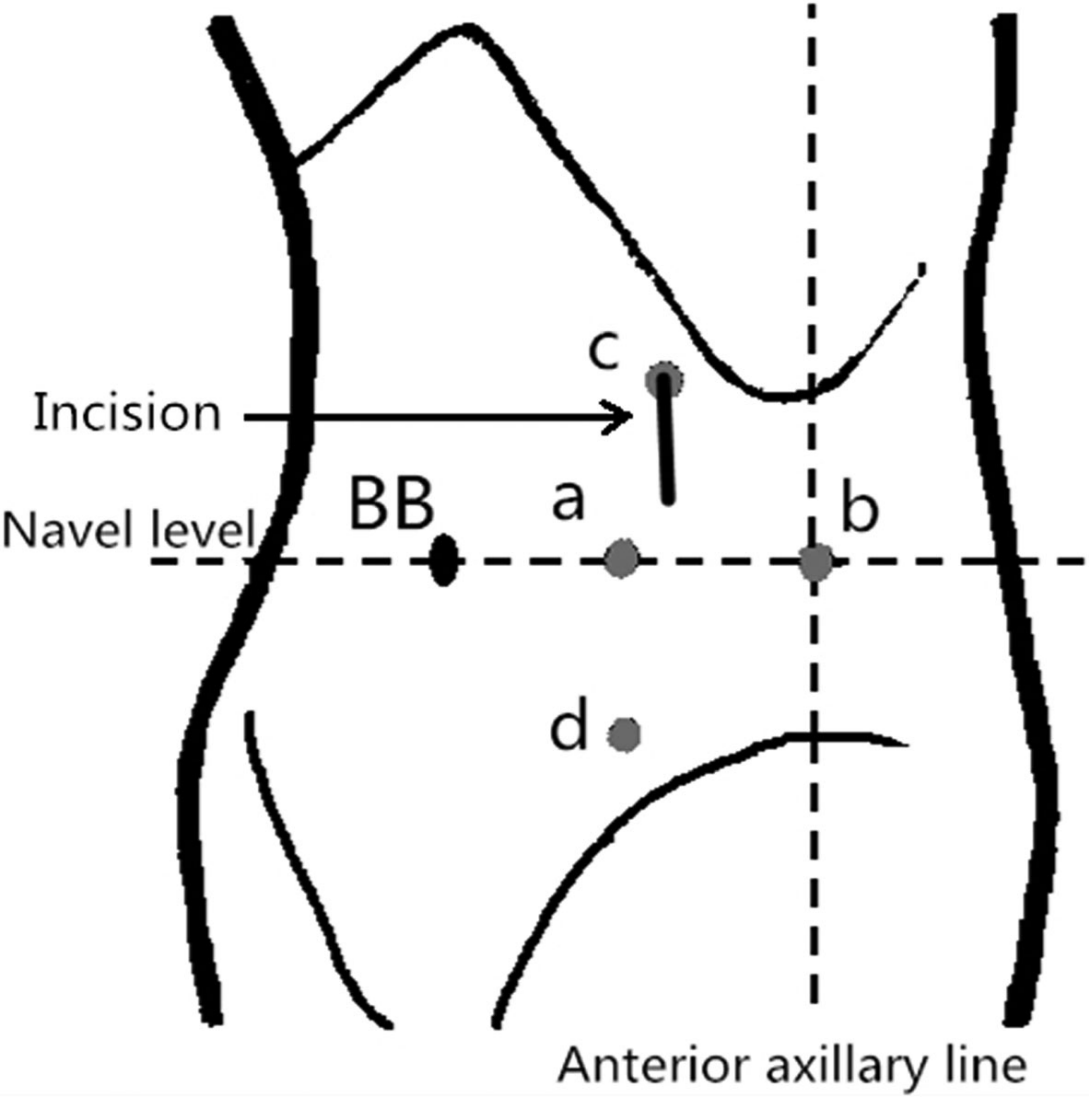
Laparoscopic ports (a–d) and incision wound of test group (for a left-side upper urinary tract urothelial carcinoma). BB, belly button

**Fig. 3:**
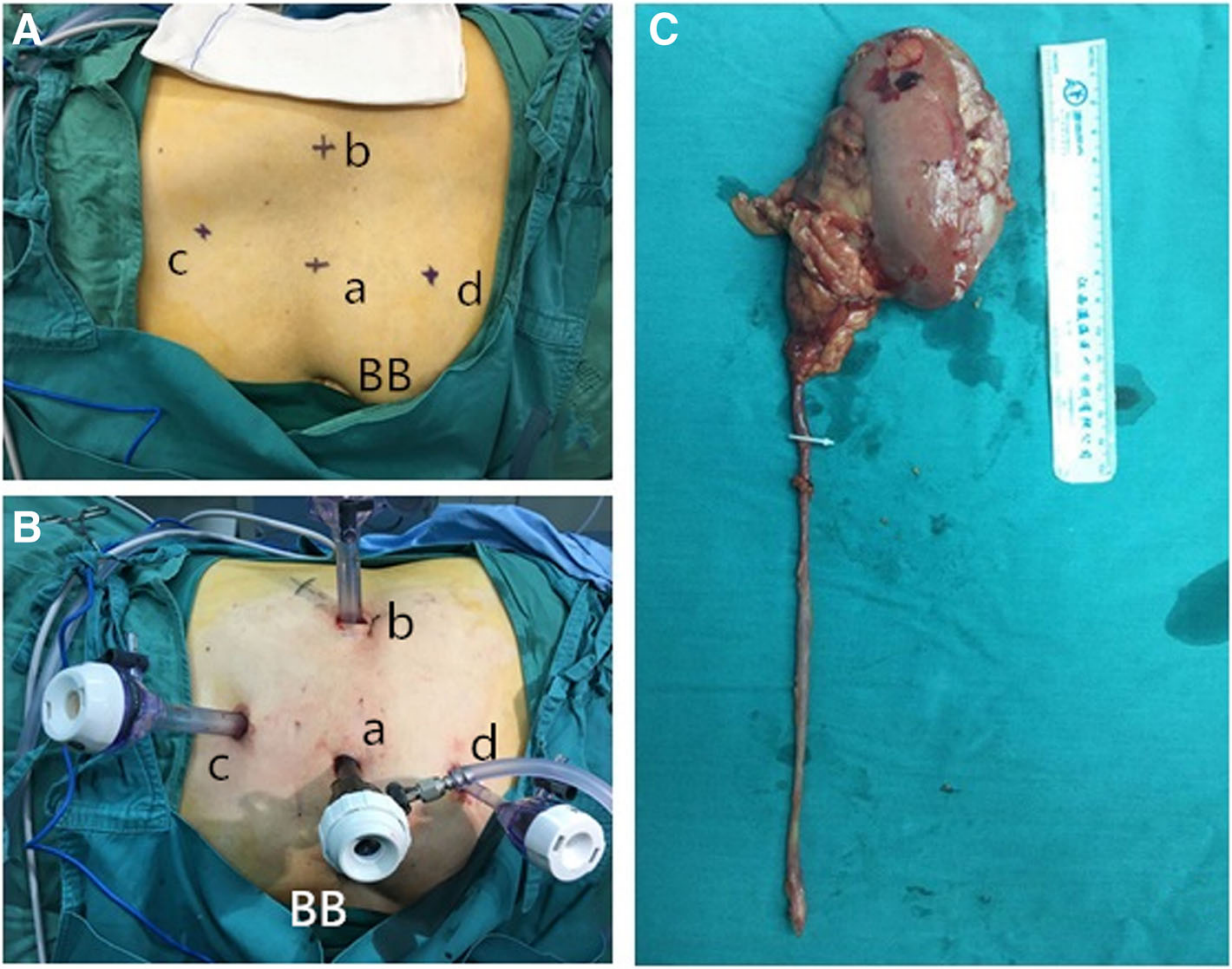
Trocar positions for a left-side upper urinary tract urothelial carcinoma (a, b) and a resected specimen (c). BB, belly button

**Fig. 4:**
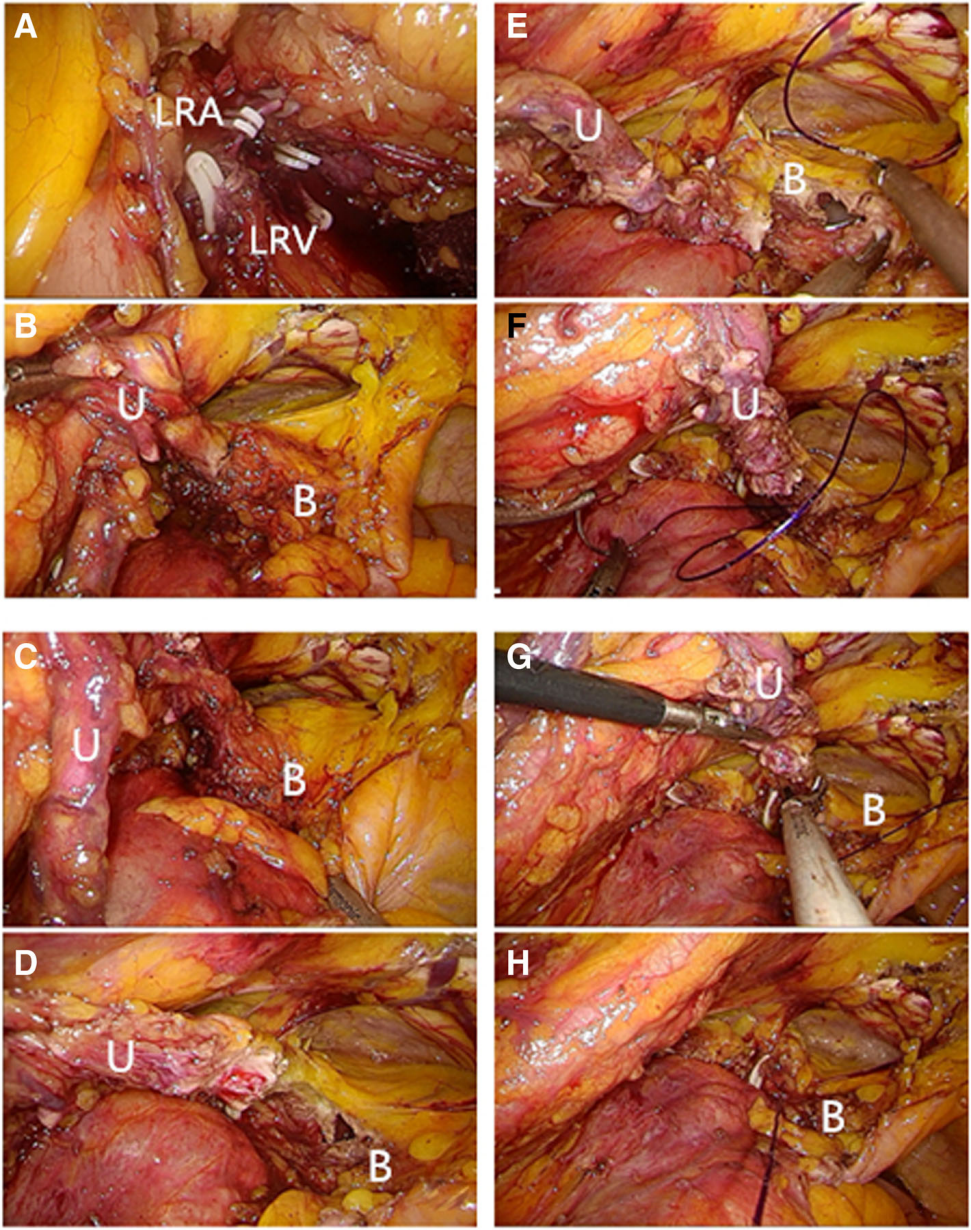
Representative images of the surgical procedures. a Ligation of the renal artery. b, c Pulling the ureter to expose the bladder. d Cutting off most of the bladder. e Pulling the ureter to assist in suturing the bladder. f Suturing most of the bladder. g Cutting off the remaining junction of the ureter and bladder. h Suturing the bladder completely. LRA, left renal artery; LRV, left renal vein; U, ureter; B, bladder

A catheter was inserted into the urethra after general anesthesia, and a surgical drape was placed below the patient’s buttocks to facilitate adjustment of the patient’s posture. The patient took a 70° lateral decubitus position with the lesion side up (an example of left-side UUTUC is shown in Fig. 2 and Fig. 3 (a)). The patient’s belly button was placed at the middle joint of the operating bed, with the waist bridge raised slightly, and the operative region routinely sterilized. The surgeon stood on the patient’s healthy side and performed the operation as well as the assistant (the operator was on the head side), the camera assistant stood in the middle facing the patient’s head, and the monitor was on the lesion side. First, a small incision was made at port a (4 cm lateral side of the navel), and towel forceps were used to clamp and lift the abdominal wall on both sides of the incision. The Veress method was used to establish the pneumoperitoneum, the pressure of which was maintained at about 14 mmHg. The 11-mm trocar was disposed into the incision as an observation hole, followed by lens insertion. The trocar (12 mm) was placed at the intersection point of the navel level and anterior axillary line (port b), another trocar (12 mm) was placed at the intersection point of the lateral border of the rectus abdominis and the plane 5 cm above the navel level (port c), and another trocar (5 mm) was placed at port d which was 5 cm below port a (Fig. 3 (b)). At this time, the operation was mainly based on port b and c trocars. If necessary, the port d trocar was used to assist the operation. After entering the abdominal cavity, the paracolic sulci and posterior peritoneum were incised with an ultrasonic scalpel to expose the renal fascia behind it. The renal fascia was opened at the mid-kidney position; the renal vein was located and freed; the reproductive vein and lumbar vein at the branch of the renal vein were located, clamped with Hem-o-lock, and cut; then, the renal artery was operated in the same way as the renal vein (Fig. 4a). The kidney was freed along its upper pole, and the adrenal gland was retained. Then, the ureter was freed to the furcation level plane of iliac vessels along the level of the kidney’s lower pole and the front of the psoas muscle. The ureter was clamped with Hem-o-lock to prevent from the spread of tumor, and the kidney was completely freed as well as most of the ureter. After inspecting the surgical wound to ensure no active bleeding, the patient’s foot was raised and the head was lowered. If the patient was overweight, the circuit nurse could change the patient’s position from 70° to 40° by dragging the surgical drape below the buttocks, and the adjustment of the patient’s position could improve the comfort level of the surgical operation. The bladder was filled through the urinary catheter preoperatively placed, which facilitated the separation of the lower ureter, the excision of the bladder cuff, and the sutures of the bladder through port b and d trocars. The operating direction of the operator was from the head side to the foot side, and the camera assistant will stand on the head side of the patient. If necessary, the laparoscopic instruments were introduced through port c to facilitate exposing the visual field. The port a trocar was still the observation port. Pulling the ureter exposed the bladder and facilitated the excision of the bladder cuff (Fig. 4b–d). Before the bladder cystectomy was basically completed, the catheter sac was properly extracted to increase the surgical operation space. The bladder was sutured and pulled first with a 2-0 barbed suture (Fig. 4e, f), and the bladder was completely sutured after cutting off the remaining junction of the ureter and bladder (Fig. 4g, h). After examination of surgical wounds for any obvious bleeding, all resected specimens were packed in self-made specimen bags and discarded (Fig. 3 (c)). The incision was extended approximately 3–4 cm downwards from port c, and a drainage tube was placed at port b. The gas which is in the abdominal cavity is released, all the trocars were pulled out, and the incisions were finally closed layer by layer.

#### Control group

**Fig. 5:**
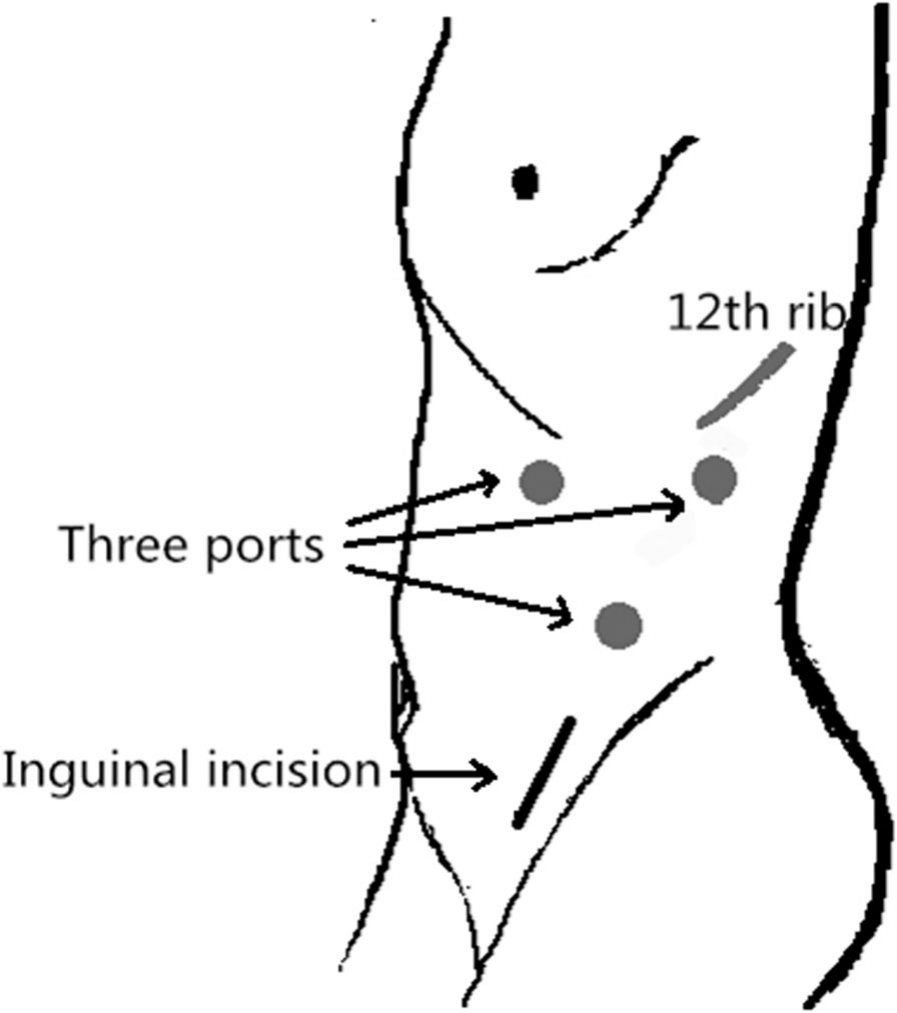
Laparoscopic ports and incision wound of control group (for a left-side upper urinary tract urothelial carcinoma)

After general anesthesia with endotracheal intubation, the patient will be placed in the lateral position and routinely disinfected and the surgical drapes are placed. The posterior abdominal cavity was established through the three-hole method (Fig. 5). The blood vessels of the renal pedicles were freed and blocked, and then the kidney was completely freed. The ureter was separated maximally, and then the distal end of the ureter will be clamped with Hem-o-lock. The patient will be changed to a supine position and re-sterilized, and the surgical drapes are placed. A 5–6-cm inguinal incision was made on the lesion side, and the remaining ureter and part of the bladder wall were removed. The bladder was sutured with a 2-0 barbed suture, and then the drainage tube was placed and the incision was closed.

### Postoperative bladder irrigation and follow-up

All patients had been followed until Oct 2018. All patients received bladder irrigation chemotherapy 2 weeks after surgery: 15~30 mg/BSA (m^2^) pirarubicin dissolved in 5% glucose injection to 500~1000 μg/ml final concentration was injected into the bladder cavity for 30 min once a week for 4~8 times and then once a month for a total of 1 year. Cystoscopy was performed approximately every 3 months for 1 year after the surgery, every 3~4 months during year 2, every 6 months for year 3~5, and annually thereafter. Patients were regularly reviewed by urinary ultrasound, chest X-ray, etc.

### Statistical analysis

The corresponding indicators of the two groups were recorded and compared, and then the data will be analyzed by using the SPSS 23.0 software. *T* test and the chi-square test or Fisher’s exact test were used for the comparison of quantitative data and qualitative data, respectively. *P* < 0.05 was considered statistically significant.

## Results

All patients successfully completed the operation, and no complications occurred such as conversion to open surgery, massive bleeding, and adjacent organ injury. And the postoperative pathology confirmed that the bladder margin of all the patients was negative. No significant difference was found in the general conditions of the patients such as age, gender, and tumor location between the two groups (*P* > 0.05). No significant difference was found in the postoperative recovery time of gastrointestinal function or tumor recurrence rate between the two groups (*P* > 0.05). However, there was significantly shorter time of operation, less intraoperative blood loss, earlier ambulation, and shorter hospitalization time for the test group compared with the control group (Table 1) (*P* < 0.05). The visual analogue scale (VAS) scores of the test group at 1 h, 12 h, and 24 h after operation were lower compared with those of the control group (*P* < 0.05). The results showed that the new surgical method could reduce surgical trauma and promote faster recovery of patients.

## Discussion

The incidence of UUTUC is much lower than the bladder urothelial carcinoma [6], accounting for about 5 to 6% of upper urinary tract tumors [7]. Urinary urothelial carcinoma has the characteristics of high recurrence rate and multi-center growth. Therefore, currently, nephroureterectomy is still the standard surgical procedure for UUTUC [8].

In recent years, with the popularization of minimally invasive intervention and advances in laparoscopic techniques, the standard surgical procedure for UUTUC has gradually shifted from traditional open surgery to laparoscopic surgery [2, 3]. Minimally invasive surgery can achieve the same therapeutic effect as open surgery and can reduce intraoperative blood loss, reduce postoperative pain, increase incision cosmetic satisfaction, accelerate patient recovery, shorten postoperative hospital stay, and reduce the occurrence rate of perioperative complications [9–11]. At present, for UUTUC, the more mature surgical procedure is retroperitoneal laparoscopic nephroureterectomy plus transurethral electric resection of the ipsilateral bladder cuff. It has been reported that transurethral electric coagulation plus retroperitoneal laparoscopic nephroureterectomy for UUTUC could reduce the shedding and implantation of tumor cells [12]. And more trials proved the benefit of retroperitoneal laparoscopic surgery in the treatment of UUTUC [13–15]. For advanced patients, some trials indicated that open surgery was better than minimally invasive surgery [16–19], and the oncologic outcomes of UUTUC between the two procedures were similar [20]. However, minimally invasive surgeries increased the anesthesia time and operation time due to the change of position and secondary disinfection during the operation, and thus increased the incidence of perioperative complications.

Nowadays, there have been many reports on the treatment of UUTUC with laparoscopy. In this report, we evaluated the effectiveness and safety of a new surgical procedure in laparoscopic nephroureterectomy for UUTUC in which the change of patient’s position was no longer needed. Compared with the conventional procedure, our procedure has the following features: (1) The anatomical positions of the trocars are relatively fixed and the establishment of them are relatively simple which can shorten the surgical learning curve. (2) This operation is performed through the abdominal approach which has clear anatomical landmarks and relatively wide operating space. (3) A good vision can facilitate a tight suture of the bladder incision, which can reduce postoperative catheter indwelling time and the length of stay. (4) There is no need for secondary sterilization during the operation. (5) The adjustment of the position of the patient can be made by dragging the surgical drape below the buttocks by the nurses, which can avoid excessive deviation of the operating axis from the lesion side, avoid the increase of operating distance, and improve the operative comfort. (6) The surgeon can complete the resection of the kidney, full-length ureter, and part of the bladder with four trocars, and no extra trocars or incisions are needed. (7) Extending the incision along the lateral border of the rectus abdominis to remove the specimens can minimize muscle damage and facilitate recovery after surgery.

In summary, total laparoscopic nephroureterectomy performed under a single surgical position was effective for the treatment of UUTUC. It had the advantages of shortening operative time and reducing intraoperative blood loss. Patients could get out of bed earlier after surgery which facilitates recovery after surgery. The new operation procedure also shortened the length of hospital stay. This new surgical treatment of UUTUC is effective, and it is worthy of clinical application and promotion.

## Conclusions

The total laparoscopic treatment of UUTUC under a single surgical position could shorten the length of stay and accelerate recovery of patients, and it is a viable surgical procedure which deserved clinical application and promotion.

## Abbreviations

CTU: Computed tomographic urography
GFR: Glomerular filtration rate
MRU: Magnetic resonance urography
UUTUC: Upper urinary tract urothelial carcinoma
VAS: Visual analogue scale
